# Thyroid hormone induces ossification and terminal maturation in a preserved OA cartilage biomimetic model

**DOI:** 10.1186/s13075-024-03326-5

**Published:** 2024-04-25

**Authors:** N. M. Korthagen, E. Houtman, I. Boone, R. Coutinho de Almeida, K. Sivasubramaniyan, R. Mahdad, R. G. H. H Nelissen, Y. F. M. Ramos, M. A Tessari, I. Meulenbelt

**Affiliations:** 1 Department Biomedical Data Sciences, Section of Molecular Epidemiology, LUMC, Einthovenweg 20, Postzone S05-P, 2333 ZC Leiden, The Netherlands; 2 Galapagos BV, Willem Einthovenstraat 13, Oegstgeest, 2342 BH The Netherlands; 3 Alrijne hospital, Simon Smitweg 1, Leiderdorp, 2353 GA The Netherlands

**Keywords:** T3, Chondrocyte, Osteochondral, Cartilage, Preserved, Lesioned, RNA-sequencing, Hypertrophy, Terminal maturation

## Abstract

**Objective:**

To characterize aspects of triiodothyronine (T3) induced chondrocyte terminal maturation within the molecular osteoarthritis pathophysiology using the previously established T3 human *ex vivo* osteochondral explant model.

**Designs:**

RNA-sequencing was performed on explant cartilage obtained from OA patients (*n* = 8), that was cultured *ex vivo* with or without T3 (10 ng/ml), and main findings were validated using RT-qPCR in an independent sample set (*n *= 22). Enrichment analysis was used for functional clustering and comparisons with available OA patient RNA-sequencing and GWAS datasets were used to establish relevance for OA pathophysiology by linking to OA patient genomic profiles.

**Results:**

Besides the upregulation of known hypertrophic genes *EPAS1* and *ANKH*, T3 treatment resulted in differential expression of 247 genes with main pathways linked to extracellular matrix and ossification. *CCDC80*, *CDON*, *ANKH* and *ATOH8* were among the genes found to consistently mark early, ongoing and terminal maturational OA processes in patients. Furthermore, among the 37 OA risk genes that were significantly affected in cartilage by T3 were *COL12A1*, *TNC*, *SPARC* and *PAPPA*.

**Conclusions:**

RNA-sequencing results show that metabolic activation and recuperation of growth plate morphology are induced by T3 in OA chondrocytes, indicating terminal maturation is accelerated. The molecular mechanisms involved in hypertrophy were linked to all stages of OA pathophysiology and will be used to validate disease models for drug testing.

**Supplementary Information:**

The online version contains supplementary material available at 10.1186/s13075-024-03326-5.

## Introduction

Osteoarthritis (OA) is a prevalent and chronic joint disease causing pain and disability, particularly among the elderly. Despite intense research efforts, no disease-modifying drug has been identified, hence patients have to face an invasive joint replacement surgery at end stage disease. As a result, OA has a major impact on the quality of life. And because of the ageing world population, the healthcare burden is expected to increase even further [1, 2].

To discover pathways underlying OA etiopathophysiological pathways, large-scale genome-wide association studies have been performed, identifying key genes robustly conferring risk to OA [3]. The role of these OA risk genes in the major causal routes to OA onset highlight aberrant articular cartilage maintenance processes in response to intrinsic and environmental challenges such as ageing. To study the role of OA risk genes in OA pathophysiology further, gene expression studies of cartilage and bone have been performed. These studies, on one hand, focused on assessing allelic expression imbalances (AEI) associated with OA risk alleles, which confirmed that OA risk alleles frequently act by changing the expression of position genes [4–6]. On the other hand, they focused on transcriptome-wide differential expression studies in both early (non-OA versus preserved-OA joint tissues) and ongoing (preserved versus lesioned OA joint tissues) OA. The non-OA versus preserved-OA studies highlighted that age-related changes in chondrocyte gene expression are marked by the differential expression of among others, *COL1A1* and *MMP13*, likely reflecting hypertrophic chondrocytes prone to OA onset. The preserved versus lesioned OA studies showed that, with ongoing OA pathophysiology, chondrocytes recuperate a detrimental growth plate morphology and undergo terminal maturation [7–9]. During this maturational process of endochondral ossification, growth plate chondrocytes start to proliferate, become hypertrophic and eventually break down cartilage matrix and undergo apoptosis to allow the transition to bone. Relevant to OA, in this respect, is that active thyroid (T3) is known to signal terminal maturation in growth plate chondrocytes, while we have previously shown by *in vitro* and *in vivo* functional follow-up studies [10–14] that upregulation of thyroid signalling makes cartilage prone to an OA disease state upon environmental challenges such as mechanical loading.

In the absence of reliable human age-related biomimetic OA models of the interacting osteochondral compartment, the exact OA-related cellular processes downstream of thyroid signalling in cartilage are, however, poorly defined. In that light, we have recently shown that T3 induces hypertrophy in our *ex vivo* human biomimetic model of OA as marked by modest upregulation of conventional hypertrophy markers matrix metalloproteinase 13 (*MMP13),* Endothelial PAS domain-containing protein 1* (EPAS1),* alkaline phosphatase *(ALPL)* and collagens *COL1A1* and *COL10A1* in cultured osteochondral explant*s* [15]. Nonetheless, expression of these genes in preserved cartilage samples is highly variable and not always robustly changing with ongoing pathophysiology [16, 17]. In order to characterize robust changes in cellular processes upon T3 exposure we here exploited our human *ex vivo* biomimetic model of OA by using the same established method and subsequently performing RNA sequencing (RNA-seq) analysis. Moreover, subsequent integration of our results with previously published genetic and transcriptome wide datasets, allowed identification of relevant markers of the OA pathophysiological process [3, 5, 16, 18–20].

## Materials and methods

### Osteochondral explant culture

Material from 20 patients who underwent total knee replacement surgery as a consequence of OA, was included in this study. Material was collected and processed anonymously under waste material protocols from local hospitals and the RAAK study protocol (ethical permission number P08.239/P19.013). No traceable data was collected, therefore no informed consent was required, in agreement with the declarations of Helsinki and Taipei.

Osteochondral explants were harvested from the macroscopically preserved areas of the condyle, washed in PBS, and cultured in chondrogenic differentiation medium as described before [15]. After 3-4 days, explants were treated for 10 days with 10 ng/ml triiodothyronine (T3, Sigma-Aldrich), matching control samples were left untreated. Medium was refreshed every 3-4 days. After the culture period, supernatant was stored at -80 degrees Celsius and a 1mm section from the middle of the explant was stored in 4% formaldehyde. Of the remaining explant, cartilage was separated from bone, snap-frozen in liquid nitrogen and stored at -80 degrees Celsius. To extract RNA, cartilage was pulverized using the Retsch MM200 bead miller under liquid nitrogen cooling with Trizol reagent. RNA was extracted using chloroform and isolated using Qiagen RNeasy Mini Kit (Qiagen GmbH, Hilden, Germany).

### RNA-seq

In total, 26 samples from 12 donors were sequenced. NEBNext Ultra II Directional RNA Library Prep Kit for Illumina was used for sample preparation. The sample preparation was performed according to the protocol "NEBNext Ultra II Directional RNA Library Prep Kit for Illumina" (NEB #E7760S/L). Briefly, rRNA was depleted from total RNA using the rRNA depletion kit (NEB#E6310). After fragmentation of the mRNA, a cDNA synthesis was performed. This was used for ligation with the sequencing adapters and PCR amplification of the resulting product. The quality and yield after sample preparation was measured with the Fragment Analyzer. Clustering and DNA sequencing using the NovaSeq6000 was performed according to manufacturer's protocols. NovaSeq control software NCS v1.7 was used. The experiments were performed at GenomeScan B.V., Leiden.

Differential gene expression analysis was performed using our in-house pipeline as follows: Resulting RNA-seq reads were aligned using GSNAP [21] against HRCh38 and abundances were estimated using HTseq count v0.11.1 [22]. Only uniquely mapping reads were used and the quality was checked using MultiQC v1.7 [23]. Principal component analysis (PCA) was performed to detect outliers. Outliers were further functionally checked in a pairwise manner by using normalized counts in edgeR and assessing the effect of removing each donor-pair on the outcome. Samples from four donors were removed based on these criteria, leaving 16 paired control and T3 samples, from 8 donors, for final analysis. Differential gene expression analysis was performed using DESeq2_1.30.0 in R version 4.0.2. Variance stabilizing transformation (VST) was applied and only genes that had at least 4 reads in 50 % of samples were included in the analysis. Differentially expressed genes were found using a pair-wise approach in a general linear model with donor and treatment as contrasts. False discovery rate (FDR) correction was applied and FDR < 0.05 was considered statistically significant. Enrichment analysis was performed using clusterProfiler_3.18.0 and the function enrichGO was applied.

### Quantitative Real-Time PCR

RT-qPCR validation was performed on independent cartilage RNA samples (*n *= 22) from 8 donors. cDNA was synthesized using Transcriptor First strand cDNA synthesis kit (Roche, Basel, Switzerland). Quantative gene expression analysis was performed for genes shown in Supplemental Table 1, using Fast Start Sybr Green Master mix (Roche Applied Science) with the QuantStudio 6 Real-Time PCR system or using the Biomark 96.96 Dynamic Arrays Fluidigm RT-qPCR platform. Gene expression was normalized using housekeeping genes SDHA, GAPDH and RSP11 by calculating the -deltaCT. Data was checked for normality using Shapiro-Wilk test and evaluation of Q-Q plots. RT-qPCR results were compared using the generalized estimating equation (GEE) with robust variance estimators to account for donor effects in SPSS v26. A linear model was used when data was normally distributed, otherwise a gamma distribution with log-link was used. A *P*-value < 0.05 was considered statistically significant.

### Histology

Histological evaluation was performed on paraffin embedded sections (5 um thickness) using hematoxylin and eosin straining (H&E) performed by pathology department and toluidine blue staining as reported previously [15]. In short, osteochondral explants were fixed in formaldehyde and decalcified using EDTA (12.5%). Following paraffin embedding, sectioning and rehydration steps, sections were incubated with toluidine blue (Sigma-Aldrich). Histological grading according to the 14-point Mankin score was performed [24, 25].

## Results

### Sample characteristics

In order to increase understanding of how T3 induces hypertrophy and/or terminal maturation and how these processes mark OA pathophysiology, we performed cartilage RNA-seq analysis of control and T3 treated human osteochondral explants that were harvested from total knee replacement surgery material. The dataset consisted of 16 paired cartilage samples from 8 donors, 6 males and 2 females and with an average age of 69.9 years (range 59-76). Histological evaluation of cartilage samples after 2 weeks culture with or without T3, confirmed that relatively preserved cartilage areas were included, with an average Mankin score of 4.2 (SD = 0.8), and there was no significant difference in Mankin score between control and T3 treated cartilage.

### Previously identified T3-induced genes related to hypertrophy

Prior to genome wide expression analyses between controls and T3 exposed human articular cartilage, we extracted differential expression of *MMP13, EPAS1, COL1A1, COL10A1 and ALPL* genes, consistently associated with hypertrophy in the past, and compared them to previously published differential expression datasets of early [20] and ongoing [16] OA pathophysiology (Table 1).

**Table 1:** RNA-seq results for known hypertrophic genes

	***FC T3***	***P-value T3***	***FC OAvsH***	***P-value OAvsH***	***FC OAvsP***	***P-value OAP***
***ALPL***	***1.71***	***n.s.***	***NA***	***NA***	***1.47***	***n.s.***
***COL1A1***	***-3.00***	***2.94 x 10***^***-03***^	***126***	***7.46 x 10***^***-22***^	***1.14***	***n.s.***
***COL10A1***	***-3.90***	***9.22 x 10***^***-04***^	***8.6***	***7.39 x 10***^***-08***^	***-1.33***	***n.s.***
***EPAS1***	***1.59***	***9.58 x 10***^***-03***^	***NA***	***NA***	***-1.17***	***3,03 x 10***^***-03***^
***MMP13***	***NA***	***NA***	***50***	***6.07 x 10***^***-18***^	***-1.12***	***n.s.***

Statistical difference for T3 RNA-seq was determined by DESeq analysis. Fold change (FC) and uncorrected P-values are shown for T3 versus control. Data for OA versus healthy (OAvsH) from Karlsson et al. [20]. Data for OA versus preserved (OAvsP) from Coutinho de Almeida et al. [16]

*N.s.* not significant, *NA* Not available

Notable in Table 1 are *COL1A1* and *COL10A1* that were significantly downregulated upon T3 exposure (FC = -3.0, *P* = 2.9 x 10^-03^ and FC= -3.9, *P* = 9.22 x 10^-4^, respectively) yet highly upregulated between non-OA versus OA (FC 126 and 8.6, respectively) and not significantly changed with ongoing OA pathophysiology (preserved versus lesioned OA). Moreover, we showed that *EPAS1* was significantly upregulated by T3 exposure (FC = 1.6 *P* = 9.6 x 10^-03^) yet significantly downregulated with ongoing OA pathophysiology. *MMP13* was very lowly expressed in preserved cartilage explants and did not change upon T3 exposure. *ALPL* was upregulated but did not reach significance due to a wider range in expression levels. Together these data suggest that T3 exposure is not manifested by upregulation of *CO10A1,* known as an early hypertrophy marker, but rather, as marked by mineralizing marker EPAS1, triggers late hypertrophy towards early maturation and mineralization.

### Differential expression analysis

**Fig. 1:**
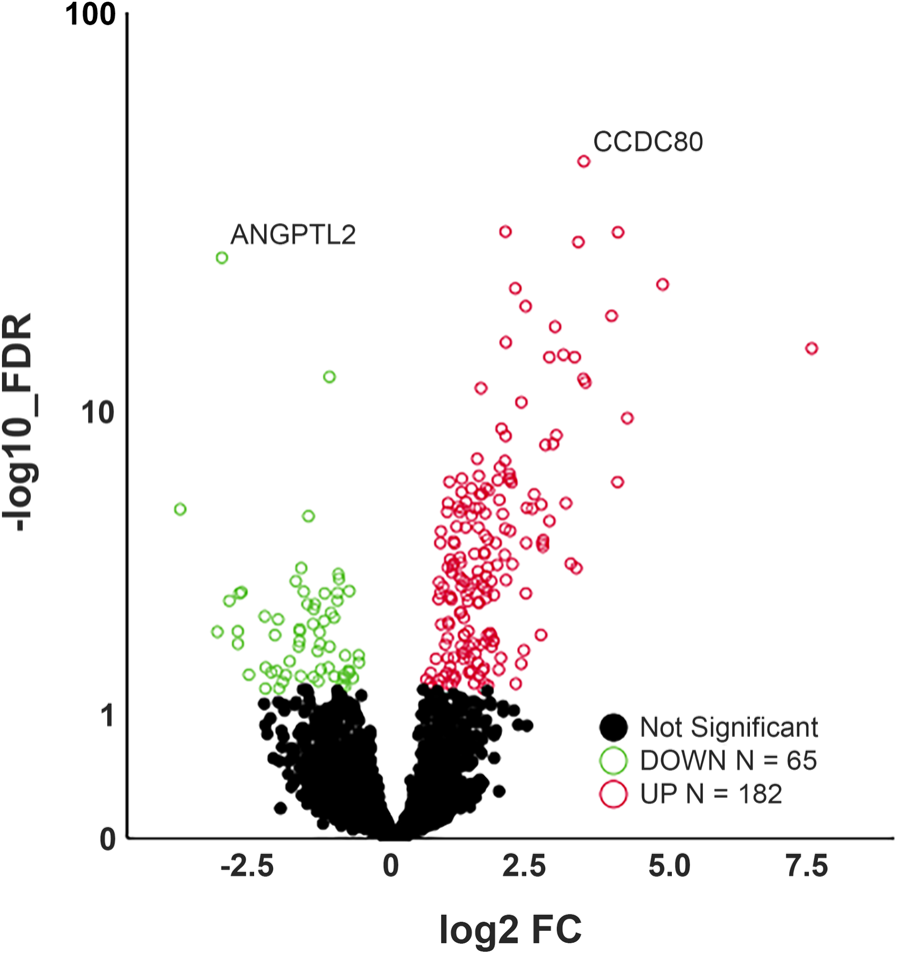
Volcano plot of differentially expressed genes after T3 stimulation. Circles represent all expressed genes in OA explant cartilage with genes significantly differentially expressed after T3-induced hypertrophy indicated in red (up) or green (down) with an FDR > 0.05. X-axis represents fold change (FC) of gene expression in T3-treated compared to control cartilage

In our RNA sequencing dataset, 10.060 genes exceeded the gene count threshold (VST > 4 counts) in at least 50% of the samples and could be included in the differentially expression (DE) analysis. Genome wide analysis with T3 exposure resulted in 247 FDR significant DE_T3_ genes (Supplemental Table 2). Of these DE_T3_ genes, 182 were upregulated and 65 were downregulated (Fig. 1).

**Fig. 2:**
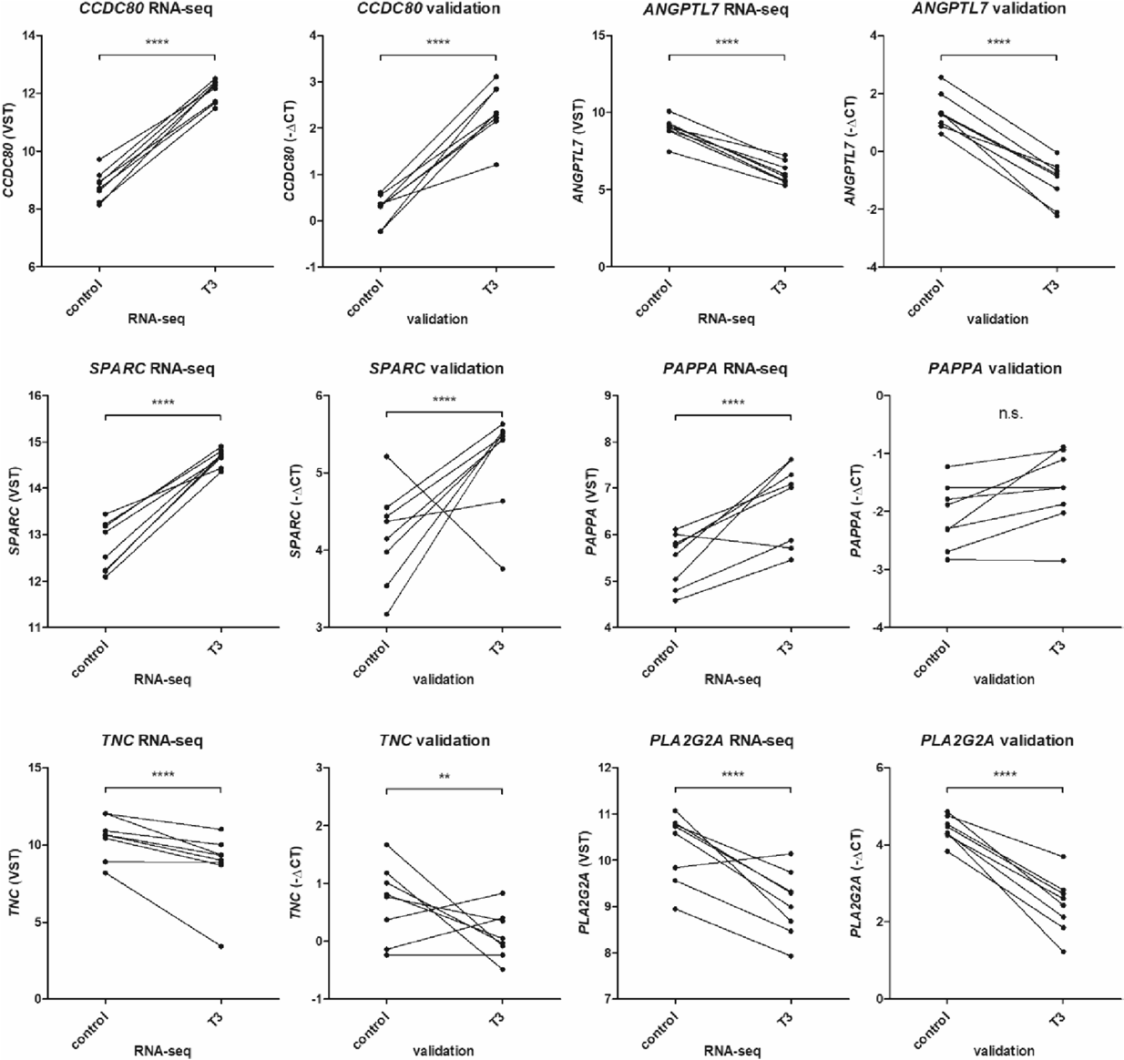
Gene expression in control and T3 stimulated explant cartilage, figure shows connected paired samples. VST data from RNA-seq analysis (paired samples from *n *= 8 donors). Statistical difference determined by DESeq analysis and with application of false discovery rate (FDR) correction. Validation data shown as -ΔCT values obtained by RT-qPCR analysis (paired samples from 8 donors). Statistical difference reflects linear generalized estimation equation (GEE). ** < 0.01;**** < 0.0001; n.s = not significant

The most significantly upregulated gene was *CCDC80*, coiled-coil domain containing 80, also known as URB (FC = 9.7, FDR = 1.3 x 10^-43^ ), predicted to bind to glycosaminoglycans and associated with cartilage extracellular matrix development and pre-hypertrophic chondrocytes [26]. The most significantly downregulated gene was *ANGPTL7* (FC = -8.1, FDR = 2.4 x 10^-25^), also known as CDT6, a member of the angiopoietin-like family. Notable among the differential 247 genes were *COL2A1* and *ACAN* as important extracellular matrix (ECM) components that were significantly upregulated after T3 treatment (FC = 6.4, FDR = 8.2x10^-8^ and FC = 1.5, FDR = 1.2 x 10^-3^, respectively), likely reflecting increased metabolic activity. As shown in Supplemental Table 3 and Fig. 2, differential expression of a selection of genes was confirmed by RT-qPCR in an independent set of 22 cartilage samples from 8 donors consisting of 4 males and 4 females with an average age of 63.3 years (range 50-81).

To identify genes that are directly downstream of T3, the presence of thyroid hormone receptor response elements in the 247 differentially expressed genes was retrieved using TFLink [27] and is shown in Supplemental Table 4. The results indicate that a substantial number of the DE_T3_ genes contain THRA, THRB and RXRA response elements, including the most upregulated genes *CCDC80* and *MLXIPL*. Among the transcription factors in our dataset, KLF9 was identified as not containing these thyroid response elements indicating other signalling pathways may be involved in T3 signalling in cartilage. To identify potential upstream transcription factors (TF) regulating T3 induced gene expression, TF enrichment analysis was performed using DAVID [28, 29] (Supplemental Table 5). Although ZIC3 and HEN1 were most significant, notable was enrichment for NFKAPPAB65, critical in thyroid-specific gene regulation [30].

### Pathway enrichment analysis

**Fig. 3:**
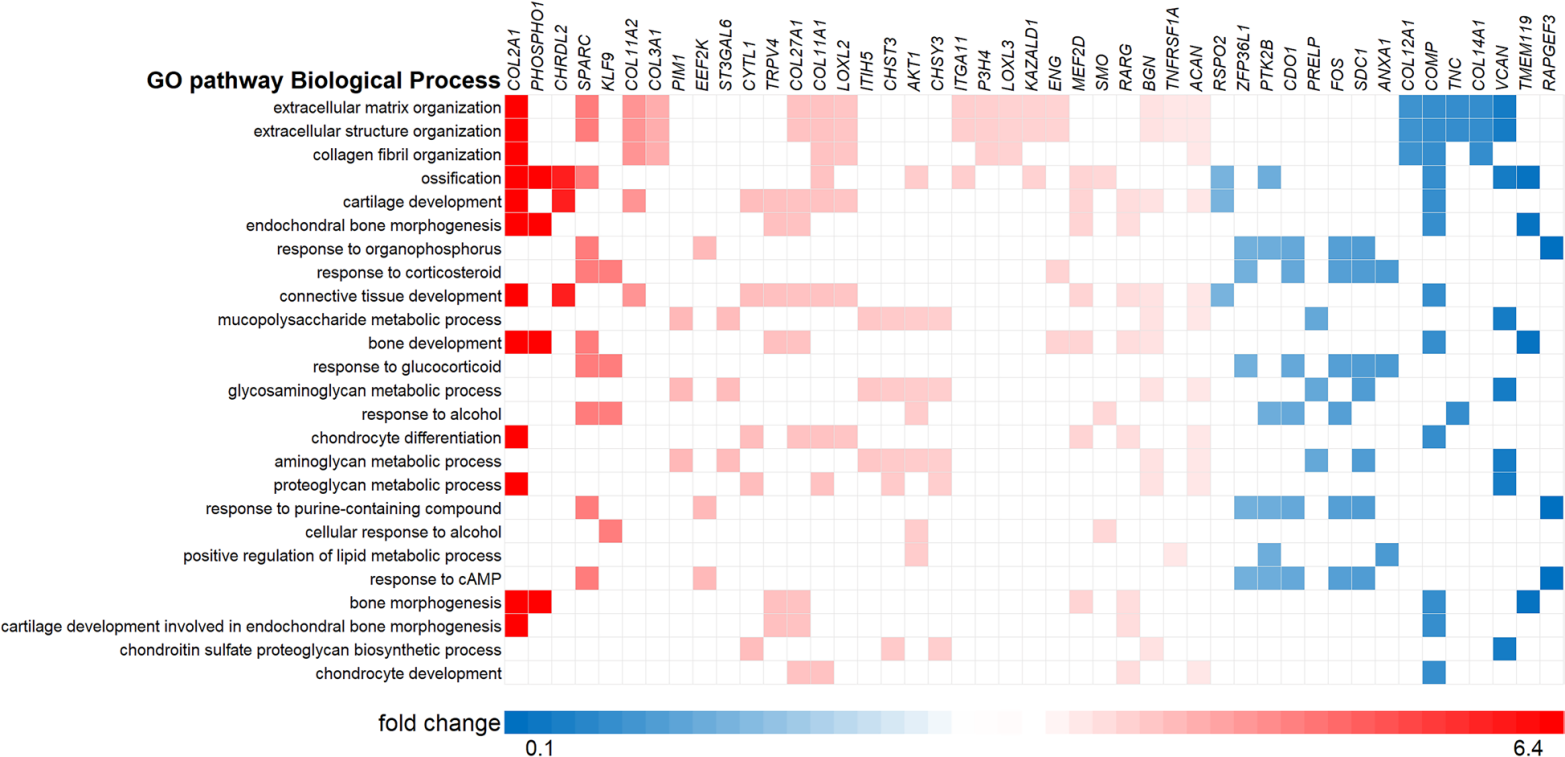
Genes present in the top 25 pathways enriched in the GO biological process subontology. Only genes present in at least 3 pathways are shown. Color reflects fold change T3 compared to control

To explore the biological pathways in which the *n* = 247 FDR significant DE_T3_ genes act, we performed ClusterProfiler analyses. As shown in Supplemental Table 6, there were 119 enriched GO pathways of which the majority (*n *= 70) was in the Biological Processes (BP) subontology. The top 25 most significant genes in the BP subontology and their most commonly associated genes are shown in Fig. 3. The two most significant pathways with the highest gene ratios in the BP subontology (both *P*_*adjusted*_ = 7.7 x 10^-16^) were ECM organization (GO:0030198) and extracellular structure organization (GO:0043062) and these pathways contained the same 34 genes (Fig. 3, Supplemental Table 6).

Notably, in these ECM pathways were also *CCDC80*, as most significantly upregulated gene, and besides several collagens, other components of cartilage ECM that were upregulated were *SPARC* (FC = 3.7, FDR = 1.1 x 10^-15^) and BGN (FC = 1.6, FDR = 1.9 x 10^-2^). In contrast, the most significantly downregulated gene, *ANGPTL7,* is involved in the expression and assembly of several ECM components such as fibronectin (*FN1*) myocilin (*MYOC*), *COL1A1* and versican (*VCAN*) and *VCAN* expression was also downregulated by T3 (FC = -4.8, FDR = 0.049), suggesting impairment of proper matrix assembly.

Additionally, Fig. 3 showed that ossification (GO:0001503) had, with 22 genes, the next highest gene ratio in the BP subontology (*P*_*adjusted*_ = 8 x 10^-6^), highlighting pathways related to organophosphorus (GO:0046683) and endochondral bone morphogenesis (GO:0060350) (see also Supplemental Table 6). Highest differential expression after stimulation with T3 for genes within these pathways was seen for *CLEC3A* (FC = 14.6, FDR = 2.9 x 10^-29^) and *SPARC*, genes that are known to be expressed in hypertrophic growth-plate cartilage. Other upregulated genes in this pathway known to be related to cartilage calcification were *ANKH* (FC = 1.9, FDR = 2.9 x 10^-4^), *PHOSPHO1* (FC = 6.4, FDR = 2.0 x 10^-14^)*, AKT1* (FC = 2.1, FDR = 2.0 x 10^-5^) and *RARG* (FC = 1.7, FDR = 5.0 x 10^-3^). Downregulation within the ossification pathway was observed for *TNFRSF11B* (FC = -3.3, FDR = 6.2 x 10^-4^)*,* encoding osteoprotegerin (OPG), the decoy receptor for TNFSF11/RANKL and TRAIL, involved in osteoclast activation. Of final note is *RSPO2,* R-spondin 2 (FC = -1.8, FDR = 1.7 x 10^-2^), a key regulator of Wnt signalling, facilitating the differentiation of proliferating chondrocytes into hypertrophic chondrocytes by enhancing Wnt/β-catenin signalling in endochondral ossification.

Together, pathway analyses suggested that chondrocyte proliferation and calcification are present in our T3 treated explants, indicating the transition to late-stage hypertrophy is accelerated by T3.

### T3 induced genes relevant for OA pathophysiology

**Fig. 4:**
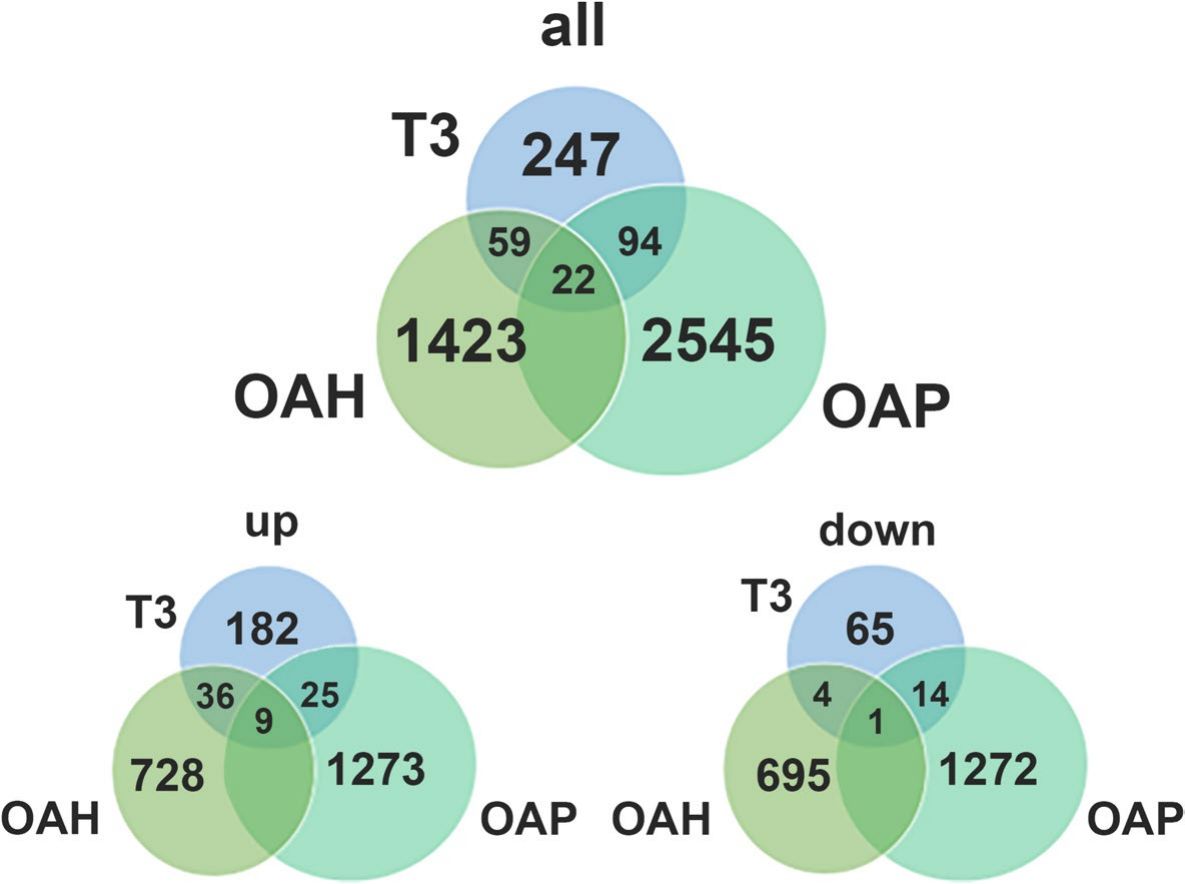
Illustrating the number of overlapping genes between T3 cartilage RNA-seq dataset, Karlsson OAH dataset [20], and RAAK OAP dataset [16]

To identify which DE_T3_ genes we identified show overlap with OA pathophysiology, we first performed a comparison with the DE gene dataset in knee joints by Karlsson *et al.* who compared early OA cartilage to cartilage from healthy individuals (DE_OAH_) [20]. We identified 40 genes with the same direction of effect in DE_T3_ and DE_OAH_ (Fig. 4, Supplemental Table 7). Notable are the upregulated *CLEC3A* and *CCDC80* as well as risk genes *COL2A1* and *SPARC*, suggesting the same activation of extracellular matrix remodelling is induced in early OA. Next we looked at the RAAK data set of paired preserved (P) and lesioned (OA) cartilage samples of OA patients (DE_OAP_) [16]. We identified 39 genes with same direction of effect in DE_T3_ and DE_OAP_, with most notable upregulated genes being again *CCDC80,* as well as *KLF2* (FC = 2.6, FDR = 1.6 x 10^-2^) and *LOXL3* (FC = 2.0, FDR = 3.8 x 10^-4^) that play a role in cartilage development. Among the genes upregulated in both DE_T3_ and DE_OAP_ were also transcription factors Hairless (HR) and Krueppel-like factor (KLF)9 (FC = 9.0 and FC = 3.8, respectively) which are known to be downstream of T3. Suggesting thyroid hormone signalling plays a significant role in OA pathophysiology.

When comparing all three transcriptome wide datasets, there were 10 genes in total with the same direction of effect in DE_T3_, DE_OAH_ and DE_OAP,_ as such marking consistent expression changes with ongoing OA pathophysiology. As already observed, *CCDC80* is one of these robustly upregulated genes, suggesting *CCDC80* is a strong quantitative marker of ongoing chondrocyte hypertrophy with OA pathophysiology. Furthermore, *ANKH* and *CDON* (FC_T3_ = 2.7, FDR = 3.6 x 10^-4^) were also found to be robustly upregulated during early, ongoing and terminally matured OA. Additionally, transcription factor *ATOH8* (FC_T3_ = -1.8, FDR = 2.8 x 10^-2^) was downregulated all three datasets and was also previously shown to be downregulated after mechanical loading [31], indicating it could be a robust marker for cartilage stress. We also identified the risk gene *PAPPA*, which is upregulated in OAP, and by T3 (FC = 3.4 and 2.7, respectively), but conversely, is significantly downregulated in OA compared to healthy cartilage (FC = -2.1).

### OA risk genes responding to T3 treatment

To investigate which OA risk genes are among the DE_T3_ genes, we compiled the genes reported as OA risk genes in genome wide association studies (GWAS) [3, 18, 19], while incorporating allelic expression imbalances of genes, to infer OA risk effects [5]. We identified 37 DE_T3_ genes as potential OA risk genes (see Supplemental Table 7), among which were many collagens as well as other cartilage matrix associated genes such as *SPARC*, *COMP*, *CHST3* and *CILP*. For most of these OA risk genes no AEI effect has been shown, some of the few exceptions being *COL11A1* (increased risk linked to an upregulation of type XI collagen; upregulated by T3 FC = 2.2 FDR = 7.7 x 10^-3^), *COL12A1* (increased risk linked to a downregulation of type XII collagen; downregulated by T3 FC = 0.35 FDR = 2.0 x 10^-3^) and *TNC* (increased risk linked to a downregulation of tenascin-C; downregulated by T3 FC = 0,31 FDR = 6.0 x 10^-3^) [4]. Other OA risk genes that fit pathological events in OA are *SMO*, encoding the protein smoothened, upregulated by T3 (FC = 1.9, FDR = 1.9 x 10^-2^), known to mediate cartilage degeneration and *PAPPA* (FC = 2.7, FDR = 5.9 x 10^-4^)*,* an important regulator of the IGF pathway. A selection of risk genes were included in the validation (Fig. 2, Supplemental Table 3). Fig. 2 shows results for risk genes *SPARC*, *PAPPA*, *TNC* and *PLA2G2A*. Upregulation of *COL27A1*, and *SPARC* was confirmed as well as downregulation of *COMP*, *PLA2G2A* and *TNC*. However, upregulation of *PAPPA* (*P*-value 0.14) and *COMP* (*P*-value 0.10) was not significant in the independent validation dataset.

## Discussion

This study aimed to elucidate the cellular processes related to hypertrophy and/or terminal maturation of articular chondrocytes with OA pathophysiology by T3 exposure to human *ex vivo* explants of preserved OA cartilage. In doing so we identified 247 DE_T3_ genes with CCDC80 as most consistently upregulated gene. Subsequent pathway enrichment analyses highlighted that DE genes upon T3 exposure were enriched within the pathways involved in extracellular matrix production and ossification, indicating acceleration of the chondrocyte transition to late-stage hypertrophy. Notable genes related to extracellular matrix, ossification and late hypertrophy included *CCDC80, SPARC, CLEC3A*, *ANKH* and *PHOSPHO1.* Upon comparing T3 responsive genes to those previously identified in early OA [20] and/or ongoing OA [16] pathophysiology we identified 10 genes, among which were *CCDC80, ANKH, CDON* and *ATOH8*, that consistently mark hypertrophy in various stages (early, ongoing, and terminal) OA pathophysiology (Fig. 4, Supplemental Table 7). We further identified relevant genes that are involved in the initiation and progression of OA hypertrophic disease stages, such as *TNC*, *SMO* and *PAPPA*. Together, we provide valuable insights into hypertrophic events in human OA articular cartilage.

Notably, *CCDC80*, the gene that was most significantly differentially expressed after T3 exposure, was also among the 10 genes that consistently mark the early and ongoing OA pathophysiological process. *CCDC80* is known to bind extracellular matrix components and is suggested to play a role in skeletal development [32]. It is increased during cartilage development in mice, where it is expressed in the ECM and in chondrocytes of different maturation stages, particularly in hypertrophic chondrocytes [26]. However, not much research has been done on *CCDC80* in human OA. Our results indicate that *CCDC80* could be a very robust and sensitive marker for hypertrophy in OA. *ATOH8* for that matter, was the only gene that was downregulated by T3 and also downregulated in the OAH and OAP datasets, and this gene is also significantly downregulated after exposure to mechanical stress [31]. Transcription factor ATOH8 (Atonal BHLH transcription factor 8), acts as a regulator of chondrocyte proliferation and differentiation in endochondral bones and is found in the region of pre-hypertrophic chondrocytes [33].

Thyroid hormone signalling pathways are complex and may involve multiple mechanisms both via nuclear and non-genomic signalling pathways [34]. Transcription factor analysis of the T3 perturbed *ex vivo* cartilage explants indicated that a large number of the DE_T3_ genes responded via other elements than the nuclear thyroid hormone response elements (Supplemental Table 4). Notable in this respect was the regulation of genes via KLF9 which has been directly linked to thyroid hormone receptor signalling [35] as well as the enrichment of transcription factors ZIC3 and NFKAPPAB65. As such they represent non-genomic pathways which mediate thyroid hormone signalling and have partial or no similarities to thyroid hormone receptors [36]. A weakness of our study is that we have not experimentally validated the nuclear and/or non-signalling e.g. via immunohistochemical data of the thyroid receptors.

To understand how T3 signalling leads to OA, the recently published GWAS study by Boer et. al. [3] provides a valuable set of genes for researchers in the OA research field to probe for genes consistently conferring risk to OA. Importantly, it was shown by functional genomic studies [4–6] that these risk alleles frequently act by changing expression of positional genes in disease relevant tissues such as cartilage and bone. Upon comparing the here identified DE genes after T3 exposure with OA risk genes, in total 37 genes were overlapping. In a few of these genes such as TNC, encoding Tenacin and COL12A1 we could subsequently compare via genome wide AEI data sets [4] the direction of effect of the risk allele in relation to the gene expression effect upon T3 exposure. For TNC and COL12A1, we showed that T3 exposure resulted in a unbeneficial downregulation of these genes e.g. preventing attempts to cartilage repair [37, 38]. Among the risk genes that are also DE after T3 exposure is PAPPA, encoding pappalysin-1. *PAPPA* was significantly upregulated by T3 and is also upregulated during ongoing OA pathophysiology. Pappalysin-1 cleaves IGFBP-2, IGFBP-4 and IGFBP-5 thereby increasing the activity of insulin growth factor IGF [39].

A large proportion of the extracellular matrix related genes that were upregulated by T3, for example *COL2A1* and *ACAN,* are considered normal, healthy constituents of cartilage. Our previous study also observed upregulation of *COL2A1* and *ACAN* in osteochondral explants after T3 treatment [15], and similar findings were observed in primary chondrocyte 3D culture [40]. Upregulation of these genes highlights that T3 causes chondrocytes to leave their maturationally arrested state, become metabolically active, and increase production of matrix proteins, possibly implicating a loosening of epigenetic control that sets them on the path towards recuperation of a growth plate-like morphology. Notably, many of the cartilage extracellular matrix related genes that are upregulated by T3 such as collagens (*COL2A1*, *COL3A1*, *COL11A2*, *COL15A1*, *COL27A1*), are risk genes for OA as well as being upregulated in early OA cartilage compared to healthy cartilage, indicating their upregulation is, indeed, not generally beneficial but might be a response to injury that triggers an anabolic rescue response, that causes the chondrocytes to leave their maturationally arrested state, leading to hypertrophy and the recuperation of a growth plate phenotype. Although it must be noted that there was no macroscopic effect on the cartilage noted at this stage, as shown by the Mankin score.

The relationship between thyroid hormone and OA has been long established but the processes involved that lead to cartilage degeneration remained unclear. It has previously been shown that risk variants of the *DIO2* gene, the catalytic activator of thyroid hormone, increase the risk of OA by regulating local activity of T3 in the cartilage [11]. Furthermore, hyperthyroidism, hypothyroidism and systemic sensitivity to thyroid hormone have also been associated with increased risk of developing OA [41, 42]. Our study indicates that CCDC80 may prove to be a useful diagnostic marker for OA that could directly link the thyroid hormone pathway and joint disease.

In the current study we have shown that T3 induced upregulation of the hypertrophy genes *EPAS1* and *ANKH*, yet resulted in downregulation of the well-known hypertrophy markers *COL10A1 and COL1A1.* Since *COL10A1* and *COL1A1* are upregulated during early OA (i.e. non-OA versus preserved OA cartilage) [20], and *EPAS1* and *ANKH* particularly in ongoing OA (i.e. preserved versus lesioned OA cartilage) [16], we suggest that *COL10A1* and *COL1A1* are markers for early hypertrophy in cartilage while the upregulation of *EPAS1* and *ANKH* is more reflective of late stage hypertrophy induced by T3. This is further confirmed by the study of van der Kraan and van den Berg (2012) which showed *COL10A1* expression was higher in less degenerated than in more degenerated areas of the cartilage [17]. Moreover, HIF-2α (encoded by the EPAS1 gene) is essential for endochondral ossification and acts by enhancing promoter activities of *MMP13* and *VEGFA* [43] whereas *ANKH* actively contributes to tissue calcification and mineralization by enhancing inorganic phosphate (Pi) concentrations and modulating osteopontin (OPN) phosphorylation [44]. Together, this confirms that T3 particularly induces late stage hypertrophy and/or terminal maturation.

## Conclusion

In conclusion, this study identified key players involved in cartilage degeneration induced by T3 and provides new insights into the role of T3 in OA pathophysiology. T3-induced changes in genes related to the extracellular matrix and ossification. Upregulation of extracellular matrix genes by T3 seems to reflect the activation of OA chondrocytes, which could initiate their recuperation to a growth plate-like morphology. Our analysis shows that there are several genes with high expression that are affected by T3 and potentially reflect late-stage hypertrophic OA disease progression, chondrocyte terminal maturation and cartilage mineralization. These results will improve the identification of chondrocyte terminal maturation in patients and the development of targeted therapeutics. Our established human OA disease model of chondrocyte hypertrophy and terminal maturation will be further exploited for preclinical studies on drug target discovery and efficacy testing.

### Supplementary Information

**Supplementary Material 1.**

## Data Availability

The main data this article are available in the article and in its online supplementary material or were derived from sources in the public domain as referenced in the article. Any further underlying data and code will be shared on reasonable request to the corresponding author.

## Abbreviations

AEI: Allelic Expression Imbalances
ALPL: Alkaline Phosphatase gene
BP: Biological Processes
CCDC80: Coiled-coil domain containing 80
COL1A1: Collagen I, alpha 1
COL10A1: Collagen X, alpha 1
DE: Differential Expression
ECM: Extracellular Matrix
EPAS1: Endothelial PAS domain-containing rotein 1
FC: Fold Change
FDR: False Discovery Rate
FN1: Fibronectin
GEE: Generalized Estimating Equations
HR: Hairless
KLF9: Krueppel-like factor 9
MMP13: Matrix Metalloproteinase 13
MYOC: Myocilin
NA: Not Available
OA: Osteoarthritis
PCA: Principal Component Analysis
Pi: Inorganic phosphate
RNA-seq: RNA sequencing
RT-qPCR: Reverse Transcription Quantitative Polymerase Chain Reaction
T3: Triiodothyronine
VCAN: Versican
VST: Variance Stabilizing Transformation
